# Editorial

**DOI:** 10.1179/2046904712Z.00000000053

**Published:** 2012-05

**Authors:** J B S Coulter

**Affiliations:** Editor-in-Chief

Paediatrics and International Child Health (PCH) is delighted to publish eight papers representing the core of the Proceedings of the Dengue Congress at the 9th International Congress of Tropical Pediatrics in Bangkok in October 2011. These papers have been excellently summarised by Usa Thisyakorn and Krisana Pengsaa in the preface to this supplement.

The resurgence and worldwide spread of dengue means that health professionals in temperate climates as well as in tropical regions need to be aware of the condition. In the former, many patients are infected whilst travelling. In sub-Saharan Africa, dengue might be overlooked in institutions overloaded by patients with severe malaria. Medical practitioners in South-east Asia have built a fine reputation in the treatment of dengue haemorrhagic fever (DHF) and dengue shock syndrome (DSS), and continue to fine-tune the World Health Organization (WHO)'s dengue case classification and management. In sub-Saharan Africa, DHF might not be recognised amongst other conditions with similar clinical features, and there is a lack of experience of treating it. This is compounded by the need for serial laboratory monitoring, e.g. haematocrit and platelet count, and, in some cases, amino-transaminases. Although there is vast experience of treating diarrhoeal dehydration, it requires less judicious monitoring than when treating DHF/DSS. Conversely, the high prevalence of malnutrition and anaemic heart failure in sub-Saharan Africa means that many paediatricians are more concerned with over-hydration, even in patients with septic shock.^1^ Also, even if required, colloids are unlikely to be readily available. In parts of the world where dengue is present but not well recognised, facilities will need to be made available for treatment, and medical practitioners and nurses trained in its management.

Over the years, the journal (previously known as Annals of Tropical Paediatrics) has taken a keen interest in dengue and, in the near future, will be publishing a number of papers and a commentary on the topic. The first issue of 2012 (volume 32) in February saw the journal relaunched with a new editorial board under the title Paediatrics and International Child Health (Paediatr Int Child Health). The rationale for the change of title has been outlined previously.^2^ Essentially, PCH is a journal of paediatrics which publishes papers mainly from developing or low-income countries. It is considered that the word ‘tropical’ no longer accurately reflects the total spectrum of the journal’s content as many of the countries from which submissions are received are not in tropical regions and therefore do not see ‘tropical diseases’, except, as evidenced above, when presented in returning travellers. The *international child health* part of the title underscores the journal’s interest in the wide spectrum of topics in community child health in developing countries which need to be brought to the attention of readers. Many non-infectious diseases, e.g. obesity, are now international disorders with an alarming increase in prevalence in low-income countries.

PCH receives papers from a wide variety of countries and it is expected that the supplement will be read by paediatricians involved in the management of dengue throughout the world. The journal shall continue to take a keen interest in dengue and its development.

## References

[1] Maitland K, Kiguli S, Opoka RD, Engoru C, Olupot-Olupot P, Akech SO (2011). Mortality after fluid bolus in African children with severe infection.. N Engl J Med..

[2] Coulter JBS (2011). Annals of Tropical Paediatrics will become Paediatrics and International Child Health from the first volume in 2012.. Ann Trop Paediatr..

